# A blubber gene expression index for evaluating stress in marine mammals

**DOI:** 10.1093/conphys/coaa082

**Published:** 2020-09-08

**Authors:** Laura Pujade Busqueta, Daniel E Crocker, Cory D Champagne, Molly C McCormley, Jared S Deyarmin, Dorian S Houser, Jane I Khudyakov

**Affiliations:** 1 Department of Biological Sciences, University of the Pacific, Stockton, CA 95211, USA; 2 Biology Department, Sonoma State University, Rohnert Park, CA 94928, USA; 3 University of Washington, Bothell, WA 98011, USA; 4 National Marine Mammal Foundation, San Diego, CA 92106, USA

**Keywords:** marine mammal, cortisol, thyroid hormone, gene expression, blubber

## Abstract

Evaluating the impacts of anthropogenic disturbance on free-ranging marine mammal populations, many of which are in decline, requires robust diagnostic markers of physiological stress and health. However, circulating levels of canonical ‘stress hormones’ such as glucocorticoids, which are commonly used to evaluate animal health, do not capture the complexity of species-specific responses and cannot be easily measured in large, fully aquatic marine mammals. Alternatively, expression of stress-responsive genes in hormone target tissues such as blubber, the specialized subcutaneous adipose tissue that can be manually or remotely sampled from many marine mammals, may be a more informative and sensitive indicator of recent (within 24 h) exposure to stressors. We previously identified genes that were upregulated in the inner blubber of juvenile northern elephant seals during experimental stimulation of the hypothalamic–pituitary–adrenal axis. In this study, we measured baseline expression levels of a subset of these genes in inner blubber of unmanipulated juvenile elephant seals of varying physiological states and correlated them with other stress markers (body condition index, corticosteroid and thyroid hormone levels). Expression of 10 genes, including those associated with lipid metabolism (*ACSL1*, *HMGCS2*, *CDO1*), redox homeostasis (*GPX3*), adipokine signaling (*ADIPOQ*), lipid droplet formation (*PLIN1*, *CIDEA*) and adipogenesis (*DKK1*, *AZGP1*, *TGFBI*), was described by three principal components and was associated with cortisol and thyroid hormone levels. Significantly, baseline gene expression levels were predictive of circulating hormone levels, suggesting that these markers may be potential indicators of exposure to stressors in marine mammal species that are inaccessible for blood sampling. A similar approach may be used to identify species-specific stress markers in other tissues that can be sampled by remote biopsy dart from free-ranging marine mammals, such as outer blubber and skin.

## Teaser

Blood hormone levels are often used to measure stress in animals, but blood cannot be sampled from many large, wild marine mammals. Gene expression in blubber, which can be sampled more easily, is correlated with blood cortisol and thyroid hormones, providing an alternative way to measure stress in marine mammals.

## Introduction

Many marine mammal populations are in decline, which may make them especially vulnerable to anthropogenic disturbance (Davidson *et al.*, 2012). Human activity in the world’s oceans continues to increase and is associated with multiple stressors (e.g. sound, chemical and physical pollution, fishing, climate change), which may have complex interactive effects on marine wildlife (Halpern *et al.*, 2015, Maxwell *et al.*, 2013, Simmonds, 2018). The mammalian physiological response to stress is mediated by the sympathetic nervous system via catecholamines, and the hypothalamic–pituitary–adrenal (HPA) axis via glucocorticoids (GCs). While adaptive in the short term, repeated or chronic stress exposure has been associated with adverse effects on reproduction, immune function, metabolism and oxidative load, among other functions (Chatelain *et al.*, 2020, Costantini *et al.*, 2011, Romero *et al.*, 2016, Sapolsky *et al.*, 2000). In marine mammals, disturbance may also cause alterations in diving and foraging behavior, potentially influencing foraging effort and success (Erbe *et al.*, 2018, Nabi *et al.*, 2018). Disturbance mitigation efforts require an understanding of the transfer functions that link disturbance to physiology and the ability to identify affected animals. Therefore, recent research priorities have focused on developing methods for assessing the physiological impacts of stressors on marine mammals (National Academies of Sciences, 2017).

Evaluating the incidence and impacts of stress in wildlife is notoriously challenging (Dickens *et al.*, 2013, Romero *et al.*, 2019). Commonly used markers, such as circulating GCs, are extremely variable and do not fully capture the complexity of the physiological response (MacDougall-Shackleton *et al.*, 2019, Romero *et al.*, 2019). For example, marine mammal responses to various stressors can be associated with significant elevation of the mineralocorticoid aldosterone and decrease in thyroid hormone levels (Champagne *et al.*, 2018, DeRango *et al.*, 2019a, DeRango *et al.*, 2019b, McCormley *et al.*, 2018, St Aubin *et al.*, 1992). Furthermore, circulating hormone measurements are not practical for many marine mammal species because they are difficult to access for blood sampling (e.g. large size, aquatic lifestyle) and, like other wildlife, are subject to the artefacts of capture stress. To address these challenges, methods for remote biopsy sampling (e.g. via a dart attached to a pole or deployed by crossbow) of marine mammals and hormone detection in a variety of other matrices, such as blubber, respiratory vapor and feces, have been actively developed (Boggs *et al.*, 2017, Burgess *et al.*, 2017, Burgess *et al.*, 2018, Champagne *et al.*, 2017, Hunt *et al.*, 2019, Noren *et al.*, 2012). However, hormone measurements often lack the sensitivity to identify recent exposure to a stressor and do not provide additional information on downstream consequences.

Corticosteroid and thyroid hormones exert their physiological effects by binding to receptors in target tissues and altering the activity of cellular proteins and genes (Brent, 2012, Sapolsky *et al.*, 2000). Glucocorticoid (GR), mineralocorticoid (MR) and thyroid (TR) hormone receptors regulate expression of hundreds of genes involved in metabolism, inflammation and endocrine signaling in mammalian adipose, a tissue that is abundant and accessible in many marine mammals (Lee *et al.*, 2014, Obregon, 2014). Changes in expression of stress-responsive genes in blubber, the specialized subcutaneous adipose tissue in marine mammals, have the potential to serve as a highly sensitive indicator of recent (within 24 h; Khudyakov *et al*., 2017) exposure to a stressor. As marine mammal blubber is vertically stratified by fatty acid composition, cellular morphology, rate of lipid accumulation and mobilization and gene expression (Ball *et al.*, 2017, Guerrero *et al.*, 2017, Struntz *et al.*, 2004), the impacts of stress hormones are likely to be more pronounced in the inner, more metabolically active layer of blubber. We previously identified a cluster of genes that were differentially expressed in inner blubber in response to acute and repeated HPA axis stimulation in a model marine mammal, the northern elephant seal (*Mirounga angustirostris*; Deyarmin *et al.*, 2019, McCormley et al., 2018, Khudyakov *et al.*, 2017). These included upregulated genes encoding metabolic and antioxidant enzymes (long-chain-fatty-acid CoA ligase 1, *ACSL1*; hydroxymethylglutaryl-CoA synthase 2, *HMGCS2*; cysteine dioxygenase 1, *CDO1*; E3-SUMO-protein ligase PIAS4, *PIAS4*; glutathione peroxidase 3, *GPX3*; microsomal glutathione S-transferase 1, *MGST1*), adipokines (adiponectin, *ADIPOQ*; leptin, *LEP*), pro-adipogenic factors (dickkopf-related protein 1, *DKK1*; zinc-alpha-2-glycoprotein 1, *AZGP1*), lipid droplet proteins (perilipin 1, *PLIN1*; cell death activator CIDE-A, *CIDEA*) and other metabolism-regulating proteins (glycine receptor subunit alpha-2, *GLRA2*), and a gene associated with extracellular matrix remodeling (transforming growth factor beta-induced, *TGFBI*), which was downregulated (Deyarmin *et al*., 2019). Gene expression changes were correlated with other markers, including increase in cortisol, aldosterone and reverse triiodothyronine (rT3) levels and decrease in total triiodothyronine (tT3) levels (McCormley *et al.*, 2018).

Many of the hormones that are responsive to stressors serve important functions in the maintenance of metabolism and show natural variations associated with daily and seasonal activities in animals. Genetic markers that co-vary with these hormones under normal (e.g. predictable and/or cyclical) activity may also be useful indicators of the response to a stressor. In this study, we evaluated the potential of these gene markers to discriminate hormonal conditions in marine mammals without the addition of exogenous stressors (i.e. natural variation within the population). We measured their expression in inner blubber along with circulating hormone levels in a cross-section of juvenile northern elephant seals of varying physiological states and identified a gene expression index comprised of 10 genes that varied with cortisol and thyroid hormone levels. We propose that baseline inner blubber expression levels of these genes may serve as sensitive and informative indicators of potential stress exposure in marine mammals and that the approach described here may be used to identify markers in other marine mammal tissues, such as outer blubber and skin.

## Methods

### Study subjects

All animal handling procedures were conducted under National Marine Fisheries Service permit 19108 and were approved by Sonoma State University and University of the Pacific Institutional Animal Care and Use Committees and the Department of the Navy’s Bureau of Medicine and Surgery. A total of 30 juvenile (0.8–1.8 year old) northern elephant seals of varying apparent body condition were sampled for the study (17 females, 12 male, one unknown). One male subject was excluded from the analyses due to an insufficient amount of sample for gene expression analyses.

### Sample collection

Baseline sample collection was conducted as previously described (Deyarmin *et al.*, 2019, McCormley *et al.*, 2018) during September to November of 2017 at Año Nuevo State Reserve, San Mateo County, CA, USA. This period is generally a short haul out and is not associated with extensive fasting, molting or reproduction. Seals were chemically immobilized using an intramuscular injection of ~1 mg/kg tiletamine-zolazepam HCl (Telazol, Fort Dodge Animal Health, USA). Sedation was maintained with intravenous doses of ketamine (0.25–1 mg/kg) (Fort Dodge Animal Health, IA, USA). Blood samples were collected within 15 ± 5 min of initial immobilization from the extradural vein using an 18 G, 3.25-inch spinal needle into vacutainer tubes (BD Life Sciences, USA) and chilled until further processing. Serum or plasma were isolated by centrifugation for 15 min at 3000 g and stored at −80°C. Blubber samples were collected within approximately 30 min of initial immobilization from the posterior flank of the animal using a 6.0-mm diameter biopsy punch (Miltex, USA). Two blubber biopsies were collected from each animal, one of which was immediately frozen in liquid nitrogen while the other was minced and incubated in RNA*later*™ Stabilization Solution (~300 mg tissue per 1.5 mL; Invitrogen, USA) with tube rocking for 24 h at 4°C, which has been shown to decrease RNA degradation in adipose tissue samples from other mammals (Hemmrich *et al.*, 2010). Blubber samples were stored at −80°C until further processing (RNA*later*™ was removed from samples prior to freezing). After collection of morphometric measurements of standard length (SL) and axillary girth (AG), seals received rear flipper tags (Dalton, Oxon, UK) and were allowed to recover from anesthesia and resume normal activity.

### Hormone assays

Serum cortisol, total T3 (tT3) and reverse T3 (rT3) were measured in duplicate using radioimmunoassays (RIAs; cortisol: cat# 07221102, tT3: cat # 0613254215, both from MP Biomedicals, USA; rT3: cat # 38-RT3HU-R125, Alpco, USA), which were previously validated for use in northern elephant seals (Ensminger *et al.*, 2014, Khudyakov *et al.*, 2017). Aldosterone was measured in duplicate using an enzyme-linked immunosorbent assay (cat# 11-ALDHU-E0, lot # 172040; Alpco, USA) previously validated for use in elephant seals (McCormley *et al.*, 2018). Mean intra-assay CVs were <2.5% for all hormone assays.

### RNA isolation

The inner half of each blubber sample (closest to muscle) was used for RNA extraction as this tissue layer is considered more metabolically active than outer blubber in marine mammals (Ball *et al.*, 2017, Struntz *et al.*, 2004). In addition, inner blubber was used for elephant seal transcriptomes that identified the markers used in this study (Deyarmin *et al.*, 2019, Khudyakov *et al.*, 2017). Blubber tissue (~100 mg) was minced with a sterile scalpel on dry ice and homogenized in 1 mL of Qiazol (Qiagen, USA) by bead beating in a Bullet Blender Storm 24 instrument (Next Advance, USA) for two 2-min cycles. Tissue homogenates were further disrupted using QIAshredder tubes (Qiagen, USA). RNA was purified using the RNeasy Lipid Mini Kit (Qiagen, USA) following the manufacturer’s protocol with a 15-min on-column DNase I digest. RNA quantity and integrity were determined using the Broad Range RNA Assay on the Qubit 3.0 Fluorometer (Life Technologies, USA) and the RNA Pico 6000 Assay on the 2100 Bioanalyzer (Agilent Technologies, USA), respectively. RNA samples had integrity values (RINs) between 7.0 and 8.6. No differences in RINs were observed between samples that were flash-frozen in liquid nitrogen and those that were preserved in RNA*later*™ solution. Therefore, tissues preserved using either method were used interchangeably for RNA isolation.

### RT-qPCR

Complementary DNA (cDNA) was synthesized from 500 ng of total RNA by reverse transcription (RT) using SuperScript IV VILO Master Mix with ezDNase (Thermo Fisher, USA). cDNA samples were diluted 1:10 and 2 μL were used in each 20 μL real-time polymerase chain reaction (qPCR) with PowerUp SYBR Green Master Mix (Thermo Fisher, USA). qPCR was performed on a QuantStudio 5 Real-Time PCR System instrument (Thermo Fisher, USA) using the following program: 2 min at 50°C, 2 min at 95°C and 40 cycles of 15 s at 95°C followed by 60 s at 60°C. All samples were run in triplicate with all intra-assay and inter-assay CVs < 0.5%. No-template and no-RT enzyme controls were included in each run and showed no amplification.

Primers for qPCR assays were designed to target genes that were previously identified as differentially expressed in elephant seal blubber in response to repeated ACTH administration (Deyarmin *et al.*, 2019). Candidate genes were selected based on three characteristics: (i) significant BlastX hits to a protein with known function in the UniProt SwissProt database, (ii) high transcript abundance (number of transcripts per million, TPM ≥ 20) and (iii) relatively consistent expression levels between biological replicates (mean CV for replicate TPM = 24.4%). Primers were designed to target highly conserved regions of differentially expressed genes using NCBI Primer-Blast. Primer sequences are shown in Table 2. All primers were used at 400 nM final concentration. Primer efficiencies (Table 2) were determined using standard curves of five 1:2 dilutions of pooled cDNA. Primer specificity was confirmed using melt curve analysis, gel electrophoresis and Sanger sequencing of qPCR products.

YWHAZ and NONO, which were used as reference genes in a previous blubber gene expression study (Khudyakov *et al.*, 2017), were evaluated for stability in expression across samples using BestKeeper algorithm (Pfaffl, 2004). YWHAZ was the most stable gene across all samples (SD = 0.47, CV = 2.06%) and was thus used as the reference gene in this study. Normalized gene expression values (delta C_T_) were calculated by subtracting the C_T_ of the gene of interest from the C_T_ of the reference gene (Huggett *et al.*, 2005, Schmittgen *et al.*, 2008). Normalized gene expression ratios and primer efficiencies were calculated using Microsoft Excel 2017.

### Statistical analyses

All statistical analyses were conducted using R v3.6.0 in RStudio v1.1.453 (R Core Team, 2016, 2019). Association between variables was evaluated using Spearman correlation (*r_S_*) analysis with Benjamini and Hochberg correction for multiple hypothesis testing (*FDR* = 0.05) using the ‘psych’ package (Revelle, 2019). Differences in putative stress markers between sexes were evaluated by one-way analysis of variance (ANOVA). Levene’s and Shapiro–Wilk tests were used to determine whether variables met equal variances and normality assumptions, respectively. Nonparametric ANOVA (Kruskal–Wallis χ^2^ test) was used for variables that did not meet ANOVA assumptions.

Body condition index (CI) was calculated as CI = (AG/SL) × 100, which has been correlated with adiposity in several species of phocid seals (Pitcher, 1986, Rea *et al.*, 2016, Ryg *et al.*, 1990). Large CI values indicate animals with higher AG, and thus potentially higher adiposity. Therefore, animals with higher CI are likely to be healthy, while those with lower CI may be experiencing nutritional stress (Le Boeuf and Crocker, 2005).

Principal components analysis (PCA) was conducted using the principal function in the ‘psych’ package (Revelle, 2019). Initial analysis was used to obtain eigenvectors for each component in the data. Three components had eigenvalues above Kaiser’s criterion of 1 and in combination explained 76% of the variance in the data; these were extracted for the final analyses. The mean of the communalities for variables after extraction was 0.76, suggesting that Kaiser’s criterion was appropriate for this dataset (communalities >0.7 for analyses with <30 samples; Field *et al*., 2012). Factor interpretability was improved using varimax rotation. Rotated factor loadings are presented in Table 3.

Rotated factor scores were used for linear regression as predictors of hormone levels. All three components were initially included in the models, after which components that did not have significant effects were removed and more parsimonious models were run. Model residuals were visually assessed for normality and homoscedasticity and variables were log-transformed if necessary. Specifically, cortisol and aldosterone were the only variables that required log transformation. All final models were assessed for presence of outliers and influential cases, which were not detected.

## Results

**Figure 1:**
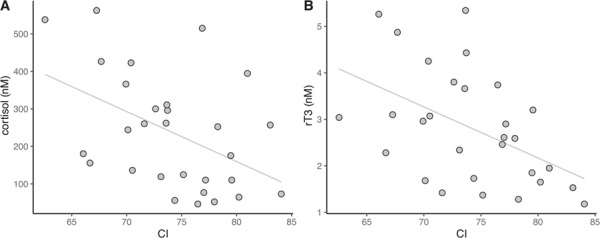
Body condition index (CI) in juvenile elephant seals (*n* = 29) was negatively associated with (A) cortisol (*r_S_* = −0.47, *P* = 0.039) and (B) reverse triiodothyronine (rT3; *r_S_* = −0.48, *P* = 0.036).

We measured biomarkers that have been associated with HPA axis activation in 29 juvenile northern elephant seals (17 female, 11 male, one unknown sex) of varying body condition, and thus varying underlying physiological states. Body CI and levels of circulating corticosteroids (cortisol, aldosterone) and thyroid hormones (tT3, rT3) were normally distributed (Shapiro–Wilk normality test, *P* > 0.05; cortisol and aldosterone were log-transformed), suggesting that the sample set adequately captured variability in a cross-sectional sample of physiological states. Mean hormone levels are shown in Table 1. CI was negatively associated with cortisol (*r_S_* = −0.47, *P* = 0.039; Fig. 1A) and rT3 (*r_S_* = −0.48, *P* = 0.036; Fig. 1B). Cortisol was negatively associated with tT3 (*r_S_* = −0.48, *P* = 0.036). None of the variables varied by sex (Kruskal–Wallis test: cortisol: *P* = 0.37, aldosterone: *P* = 0.65; ANOVA: tT3: *P* = 0.098, rT3: *P* = 0.12, CI: *P* = 0.56).

**Table 1:** Mean values of baseline circulating corticosteroid and thyroid hormone levels in 29 juvenile northern elephant seals

Hormone	Mean ± s.d.
Cortisol (nM)	237.6 ± 154.5
Aldosterone (pM)	1041.2 ± 785.3
tT3 (nM)	2.03 ± 0.61
rT3 (nM)	2.81 ± 1.21

**Figure 2:**
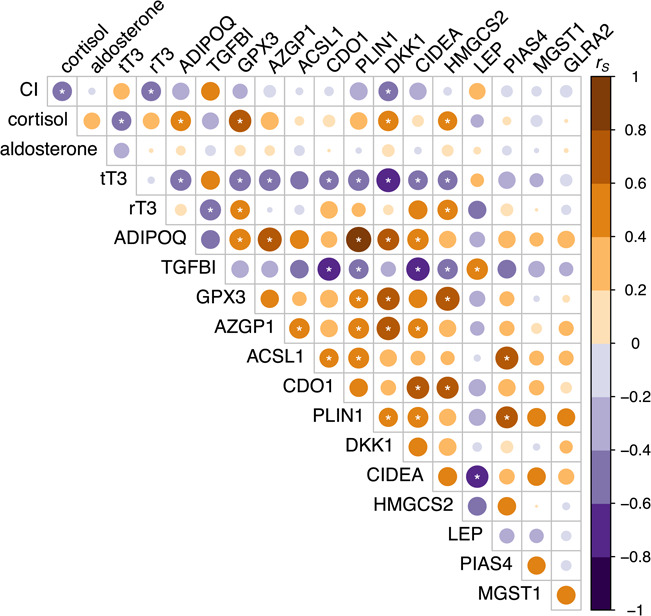
Spearman rank correlation (*r_S_*) between body condition, circulating corticosteroid and thyroid hormone levels and normalized blubber gene expression values (delta C_T_) in juvenile elephant seals (*n* = 29) of varying baseline physiological stages. Circle size denotes degree of correlation (size of *r_S_*), while circle color denotes direction of the relationship (orange: positive, purple: negative). Asterisks (*) show relationships that were statistically significant at *P* < 0.05. CI: body condition index, tT3: total triiodothyronine, rT3: reverse triiodothyronine, *ADIPOQ*: adiponectin; *TGFBI*: transforming growth factor-beta-induced protein ig-h3, *GPX3*: glutathione peroxidase 3; *AZGP1*: zinc-alpha-2-glycoprotein, *ACSL1*: long-chain fatty acid-CoA ligase 1, *CDO1*: cysteine dioxygenase type 1, *PLIN1*: perilipin 1, *DKK1*: Dickkopf-related protein 1, *CIDEA*: cell death activator CIDE-A, *HMGCS2*: hydroxymethylglutaryl-CoA synthase (mitochondrial), *LEP*: leptin, *PIAS4*: E3 SUMO-protein ligase PIAS4, *MGST1*: microsomal glutathione S-transferase 1, *GLRA2*: glycine receptor subunit alpha-2.

**Table 2:** Primer sequences and amplification efficiencies (E) of qPCR assays. All sequences are written in the 5′ to 3′ direction. Primers were designed using gene sequences from elephant seal blubber transcriptomes (Deyarmin *et al.*, 2019, Khudyakov *et al.*, 2017)

Transcript ID	Gene homolog	Protein name	Primer sequence	E (%)
TRINITY_DN592797_c2_g1	*PLIN1*	Perilipin 1	F:GCGGTCAACAAGGACCCAAC R:GGAAACACTCACAGGTGCCG	94
TRINITY_DN572974_c28_g1	*ADIPOQ*	Adiponectin	F: CCAATGTTCCCATTCGCTTTAC R: CATTCCTGGGCTGTACTACTTC	93
TRINITY_DN581259_c1_g6	*LEP*	Leptin	F: ACAGGACCAAAGCCACAGGA R:GCGAGGCCTGAGAAGCACAT	104
TRINITY_DN585354_c4_g5	*DKK1*	Dickkopf-related protein 1	F: CCAAGATCTGTAAACCTGTCCTCR: CACAGTAACAGCGCTGGAATA	103
TRINITY_DN571555_c4_g2	*GPX3*	Glutathione peroxidase 3	F: CCGGACGGTGTACCCATCAT R: GGCAGGTCTGATTTACTGCCC	107
TRINITY_DN587426_c4_g1	*CIDEA*	Cell death activator CIDE-A	F: CGCGTTGCCAATCTAGACGC R: TCCGTATCCACCACGGTTCC	100
TRINITY_DN560507_c9_g1	*HMGCS2*	Hydroxymethylglutaryl-CoA synthase 2	F:TGATGTTCAGTGACTTCCTGTC R: TGTAGGTTTCTTCCAGCGTTAG	100
TRINITY_DN591927_c4_g2	*CDO1*	Cysteine dioxygenase 1	F:TGACAAATTCCTGTCCGATAAGT R:TTCTGCCCTCCCTTTCATTAC	97
TRINITY_DN591034_c2_g1	*TGFBI*	Transforming growth factor beta-induced	F:TCCTGAAGGGGGACAATCGC R: GACCCCTTCCCTGTTGAGCA	97
TRINITY_DN571072_c3_g3	*ACSL1*	Long-chain-fatty-acid CoA ligase 1	F:GTAGCGATGGTGCTCGGAGA R: ACTTGACACCTGGATGCCCC	97
TRINITY_DN587150_c2_g2	*AZGP1*	Zinc-alpha-2-glycoprotein 1	F: TCGTTCTCCCAGTCCTCTATTC R:CCTACCTCAATGACCAGGATTTC	94
TRINITY_DN551341_c9_g1	*GLRA2*	Glycine receptor subunit alpha-2	F: GTCTCCAGACAACACAAGGAG R: CCCATCCCATAACCACTGAAA	107
TRINITY_DN587322_c7_g1	*PIAS4*	E3-SUMO-protein ligase PIAS4	F:GCGGACTTAAACACGAACTTGTCA R: GAGCTCTTCTTGGCGTAGCG	106
TRINITY_DN592891_c7_g1	*MGST1*	Microsomal glutathione S-transferase 1	F: TTAACGACTGAGCCACCCAGG R:GCATCAAGATGAACCACAAGTTGG	100
TR6987 c5_g2 (from Khudyakov *et al*., 2017)	*NONO*	Non-POU domain-containing octamer-binding protein	F:GAGGAAGGTTTCGGACTGTAAG R:GCGGAGATTGCCAAAGTAGA	95
TR33370 c1_g1 (from Khudyakov *et al*., 2017)	*YWHAZ*	14-3-3 protein zeta/delta	F: AGCAGAGAGCAAAGTCTTCTATT R: GACTGATCCACAATCCCTTTCT	100

**Table 3:** Eigenvalues and percent of explained variance for varimax-rotated components (RC1, RC2, RC3) and loading scores for 10 stress-associated genes. Loadings >0.5 are shown in bold

	**RC1**	**RC2**	**RC3**
Eigenvalue	2.37	2.73	2.52
% of variance	24	27	25
**Gene**	**Varimax-rotated factor loadings**		
*ADIPOQ*	**0.55**	0.03	**0.73**
*TGFBI*	−0.11	**−0.83**	−0.25
*GPX3*	**0.87**	0.33	0.11
*AZGP1*	0.45	0.17	**0.64**
*ACSL1*	−0.06	0.32	**0.75**
*CDO1*	0.15	**0.87**	0.15
*PLIN1*	0.23	0.27	**0.80**
*DKK1*	**0.77**	0.10	0.40
*CIDEA*	0.24	**0.73**	0.33
*HMGCS2*	**0.61**	**0.66**	0.04

**Table 4:** Parameters for linear regression models of rotated components and baseline circulating levels of cortisol, tT3 and rT3. Cortisol was log-transformed to meet the multivariate normality assumption of linear regression models. *B*: unstandardized beta, *SE B*: standard error of unstandardized beta, β: standardized beta

Coefficient	*B*	*SE B*	*t*	β	*P*
*log(cortisol)*					
Intercept	2.27	0.050	45.23		<0.0001
RC1	0.19	0.051	3.70	0.58	0.00097
*tT3*					
Intercept	2.03	0.07	27.57		<0.0001
RC1	−0.36	0.07	−4.79	−0.59	<0.0001
RC2	−0.17	0.07	−2.29	−0.28	0.031
RC3	−0.27	0.07	−3.60	−0.44	0.0014
*rT3*					
Intercept	2.81	0.18	15.30		<0.0001
RC2	0.72	0.19	3.87	0.60	0.00063

PCA was used to reduce dimensionality of the gene expression dataset. This approach was deemed appropriate using several diagnostic criteria (Field *et al.*, 2012). First, examination of the correlation matrix of dC_T_ values for 14 genes identified four (*MGST1*, *GLRA2*, *PIAS4*, *LEP*) that were removed from subsequent analyses due to having two or fewer significant (*P* < 0.05) correlations with other gene markers (Fig. 2). Bartlett’s test of sphericity indicated that the correlations between the remaining 10 genes were sufficiently large for PCA (*χ^2^*_45_ = 167.43, *P* < 0.0001). There was no evidence of multicollinearity as the determinant of the correlation matrix was 0.00089, which is greater than the recommended minimum of 0.00001 (Field *et al.*, 2012). Sampling adequacy was assessed using the Kaiser–Meyer–Olkin (KMO) measure. The overall KMO was 0.73 (values between 0.7 and 0.8 are considered ‘good’; Field *et al*., 2012), and KMO values for individual items were all >0.63. Baseline gene expression levels of *ADIPOQ*, *TGFBI*, *GPX3*, *ACSL1*, *AZGP1*, *CDO1*, *PLIN1*, *DKK1*, *CIDEA* and *HMGCS2* were described by three varimax-rotated principal components that had eigenvalues of >1 and in combination explained 76% of the variance in the data (Table 3). *GPX3* and *DKK1* loaded uniquely onto rotated component 1 (RC1). *TGFBI*, *CDO1* and *CIDEA* loaded uniquely onto rotated component 2 (RC2). *AZGP1*, *ACSL1* and *PLIN1* loaded uniquely onto rotated component 3 (RC3). *ADIPOQ* loaded onto both RC1 and RC3, while *HMGCS2* loaded onto both RC1 and RC2 (Table 3).

**Figure 3:**
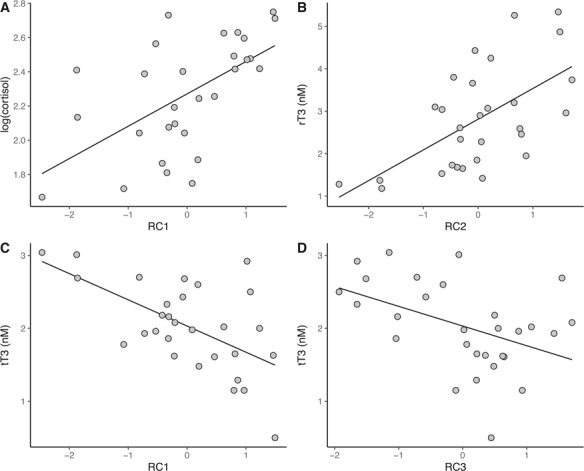
Significant associations between the stress-responsive blubber gene expression index (varimax-rotated components RC1, RC2, RC3) and hormone values in juvenile elephant seals (*n* = 29) of varying baseline physiological states were evaluated using multiple linear regression (cortisol: *F*_3,25_ = 5.16, *P* = 0.0065, *R*^2^ = 0.38; tT3: *F*_3,25_ = 13.71, *P* < 0.0001, *R*^2^ = 0.62; rT3: *F*_3,25_ = 7.13, *P* = 0.0013, *R*^2^ = 0.46; model parameters are shown in Table 4).

We next evaluated the potential of the stress-responsive gene expression index to predict circulating hormone levels. RC1 explained 33.7% of the variance in circulating cortisol levels (*F*_1,27_ = 13.71, *P* = 0.00097; Table 4, Fig. 3). There was no association between cortisol and RC2 (*P* = 0.61) or RC3 (*P* = 0.22). RC2 explained 35.6% of the variance in circulating rT3 levels (*F*_1,27_ = 14.95, *P* = 0.00063; Table 4, Fig. 3). There was no relationship between rT3 and RC1 (*P* = 0.071) or RC3 (*P* = 0.26). All three components explained a total of 62.2% of the variance in circulating tT3 levels (*F*_3,25_ = 13.71, *P* < 0.0001; Fig. 3), but the effect sizes ranked as RC1 > RC3 > RC2 (standardized beta, β = −0.59, −0.44, and − 0.28, respectively; Table 4). Therefore, the effect of RC2 on tT3 levels was negligible, explaining only 7.8% of the variance in the data.

There were no significant associations between the components and aldosterone (*P* = 0.25) or CI (*P* = 0.36). Aldosterone was not associated with any of the gene markers, while CI was negatively associated with *DKK1* (*r_S_* = −0.49, *P* = 0.036). RC3 varied with sex and was higher in males than females (ANOVA: *F*_2,26_ = 9.47, *P* = 0.0008). RC1 and RC2 were not associated with sex (ANOVA; RC1: *P* = 0.69, RC2: *P* = 0.31).

## Discussion

In this study, we evaluated whether circulating levels of aldosterone, tT3 and rT3 and expression of a suite of previously identified stress-responsive genes (Deyarmin *et al.*, 2019) may be more informative than baseline cortisol levels alone in assessing stress state in marine mammals. We measured levels of these markers in a cross-sectional sample of elephant seals of differing physiological condition evidenced by variability in body condition and hormone levels. These stress-responsive genes included several known targets of glucocorticoid and thyroid hormones and encoded adipokines, lipid metabolism enzymes and factors that regulate adipogenesis. We found that that baseline blubber expression of 10 genes—*ADIPOQ*, *ACSL1*, *HMGCS2, AZGP1*, *PLIN1*, *CIDEA, GPX3*, *CDO1*, *DKK1* and *TGFBI*—was predictive of circulating levels of cortisol and thyroid hormones but not aldosterone or body condition. Gene expression data were collapsed into three principal components that significantly varied with circulating hormone levels. We propose that RC1 represents an index of GR (and potentially TR) target gene expression that reflects increased cortisol and decreased tT3 levels and potential consequent alterations in adipogenesis, lipid metabolism, insulin sensitivity and redox homeostasis. RC2 represents an index of pro-adipogenic gene expression that is associated with elevated rT3. Finally, RC3 represents an index of gene expression associated with reduced tT3 levels and consequent potential alterations in lipid homeostasis in marine mammals.

RC1, which represented expression of the antioxidant enzyme *GPX3* and pro-adipogenic factor *DKK1*, was positively associated with cortisol and negatively associated with tT3. Cortisol induces differentiation of pro-adipocytes into mature adipocytes, stimulates lipolysis and promotes insulin resistance (Lee *et al.*, 2014), while T3 stimulates proliferation and inhibits differentiation of pro-adipocytes and enhances both lipogenesis and lipolysis in mammals (Carmean *et al.*, 2014). Suppression of thyroid hormone levels during stress and a negative relationship between cortisol and T3 have been reported in several terrestrial and marine mammal species (Atkinson *et al.*, 2015, Helmreich *et al.*, 2011). Expression of *GPX3* and *DKK1*, which are both known GR target genes, is associated with adipogenesis in rodents and humans (Christodoulides *et al.*, 2006, Lee *et al.*, 2008). *GPX3* is also associated with insulin sensitivity and protection from oxidative damage and inflammation in adipose tissue (Christodoulides *et al.*, 2006). A recent study found that *DKK1* was upregulated in human adipocytes expressing a mutant form of thyroid receptor (TRβ), suggesting that it may be directly inhibited by T3 signaling (Liu *et al.*, 2015). Upregulation of RC1, in conjunction with elevated cortisol and decreased tT3, may promote maturation of adipocytes, potentially in order to sustain elevated rates of lipid metabolism during stress.

RC2, representing expression of *CIDEA*, *CDO1* and *TGFBI*, was positively associated with rT3, a product of thyroxine (T4) deiodination by type III deiodinase (D3) that is thought to be biologically inactive. Reverse T3 was elevated and correlated with cortisol during responses to capture stress in belugas and ACTH administration in elephant seals (Champagne *et al.*, 2015, Ensminger *et al.*, 2014, St Aubin *et al.*, 1992). Interestingly, *D3* was upregulated in proliferating rat adipocytes, suggesting that rT3 production may also play a role in adipogenesis (Carmean *et al.*, 2014). *CIDEA* encodes a lipid droplet protein that is associated with insulin sensitivity and adipose expansion in humans (Abreu-Vieira *et al.*, 2015). *CDO1* encodes an enzyme in the taurine biosynthesis and cysteine degradation pathways that has been implicated in adipogenesis (Deng *et al.*, 2015). *TGFBI* encodes an extracellular matrix protein that was recently identified as a negative regulator of adipogenesis in human cells (Zhang *et al.*, 2019). *TGFBI* was downregulated in response to repeated HPA axis stimulation in elephant seals (Deyarmin *et al.*, 2019) and was negatively associated with rT3 in this experiment. Another gene, *HMGCS2*, which encodes a ketogenic enzyme that regulates fatty acid oxidation in a variety of cell types (Sikder *et al.*, 2018), loaded onto both RC2 and RC1. It is possible that the association between RC2 gene expression and rT3 levels is indirect, being driven primarily by the coincident decrease in tT3 and increase in cortisol. However, RC2 explained only 7.8% of the variance in tT3 levels and *HMGCS2* was the only gene that loaded onto both cortisol-associated RC1 and rT3-associated RC2. Recent research suggests that rT3 may bind to non-genomic TR isoforms and alter cytoskeletal dynamics (Davis *et al.*, 2008); it is possible that it may have other biological functions. An increase in RC2 expression and rT3 levels may serve to enhance adipogenesis and fat catabolism during stress.

RC3, which encompassed expression of *AZGP1*, *ACSL1* and *PLIN1*, was negatively associated with tT3. A decrease in levels of tT3, which is associated with lipogenesis (Mullur *et al.*, 2014), coincident with increased cortisol levels suggests an enhanced capacity for lipid catabolism during stress. *AZGP1* promotes lipolysis and inhibits lipogenesis in mice and humans (Bing *et al.*, 2010). *ACSL1* is an enzyme involved in fatty acid uptake, beta-oxidation and reesterification (Ellis *et al.*, 2010). *PLIN1* regulates fat droplet homeostasis and facilitates hormone-stimulated lipolysis (Kimmel *et al.*, 2016). *ADIPOQ*, which loaded onto both RC1 and RC3, is associated with insulin sensitivity, adipose tissue expansion and fatty acid oxidation (Stern *et al.*, 2016). Expression of *PLIN1* and *ADIPOQ* is indirectly regulated by thyroid hormones (Liu *et al.*, 2012, Liu *et al.*, 2015). Upregulation of RC3 and decrease in tT3 may potentially contribute to increased rates of lipolysis and fatty acid oxidation during stress.

The association of RC3 with sex was surprising given that tT3 levels measured in this study did not vary by sex. However, previous studies of this age class of elephant seals found that T3 and T4 were higher in males compared to females and were associated with differences in field metabolic rate between sexes (Jelincic *et al.*, 2017, Kelso *et al.*, 2012). It is possible that the small sample size and variability in physiological state among individuals used in this study masked observable sex differences in hormone levels. However, RC3 values were higher in males in this study and were significantly associated with tT3, suggesting that blubber gene expression measurements, which are more sensitive than circulating hormone measurements, may reflect true underlying sex differences in thyroid hormone production and signaling in marine mammals.

We found no association between gene expression and aldosterone in this study. Aldosterone, a secretagogue of ACTH, was one of the most significantly altered hormones in the repeated ACTH administration experiment (McCormley *et al.*, 2018) and has been shown to be elevated in response to external stressors in several marine mammal species besides elephant seals (Champagne *et al.*, 2018, DeRango *et al.*, 2019a, DeRango *et al.*, 2019b). Aldosterone is primarily known for its osmoregulatory and vascular functions, but recent studies have suggested that it may have additional roles in adipogenesis and insulin sensitivity (Marzolla *et al.*, 2012). However, the biological effects of aldosterone are mediated by MR, which also binds GCs with high affinity, suggesting that MR signaling in adipose tissue may be mediated in part by GCs (Gomez-Sanchez *et al.*, 2014). Moreover, mean baseline aldosterone concentrations measured in this and other elephant seal studies were 200- to 300-fold lower than cortisol concentrations (McCormley *et al.*, 2018). Therefore, any changes in blubber gene expression that may reflect aldosterone signaling are likely masked by the effects of cortisol.

Body CI was negatively associated with cortisol and rT3, which is consistent with a previous study of juvenile elephant seals that found that T4 levels significantly decreased over a fasting period during which animals lost 17% of their body fat (Kelso *et al.*, 2012). Cortisol was also negatively associated with adiposity in lactating adult female elephant seals (Fowler *et al.*, 2018), but not in juveniles (Kelso *et al.*, 2012), highlighting the importance of evaluating stress state in the context of life history stage, especially in fasting-adapted marine mammals. Body condition was also negatively associated with *DKK1*, but not with any of the other gene markers, suggesting that this gene may be a novel molecular marker of adiposity in marine mammals. Serum levels of DKK1 were also correlated with adiposity in diabetic humans, although the relationship was positive (Ali *et al.*, 2019). This difference may be due to a mismatch between mRNA expression in adipose and circulating protein levels, or the fact that high adiposity and insulin resistance are considered pathological in humans, but healthy in fasting-adapted mammals (Houser *et al.*, 2013). While CI is an approximation rather than a direct measurement of adiposity, our data suggest a potentially informative relationship between this metric and stress-associated hormones and one of their target genes. More accurate determination of adiposity (e.g. using the isotopic dilution method) will be necessary to validate these associations.

In summary, we have shown that baseline expression of 10 genes, which can be measured in marine mammal blubber by RT-qPCR, was strongly associated with other markers of stress in elephant seals. These genes encode proteins that regulate adipogenesis, fatty acid oxidation, redox homeostasis and insulin sensitivity, providing information about the functional consequences of stress-induced hormone changes in fasting-adapted marine mammals. Such consequences may include alterations in lipid homeostasis that disrupt the ability of capital breeding marine mammals to complete key life history stages that require fasting. Significantly, baseline gene expression levels in inner blubber predicted circulating cortisol and thyroid hormone levels, suggesting that these genes may be sensitive indicators of stress exposure in marine mammal species that are inaccessible for blood hormone measurements. Furthermore, the approach described here can be used to identify species-specific stress markers in other matrices, such as skin and outer blubber, which are more commonly sampled by remote biopsy in many free-ranging marine mammals.
